# The effect of customer satisfaction on parcel delivery operations using autonomous vehicles: An agent-based simulation study

**DOI:** 10.1016/j.heliyon.2022.e09409

**Published:** 2022-05-11

**Authors:** Mohammad A. Shbool, Ammar Al-Bazi, Rami Al-Hadeethi

**Affiliations:** a Industrial Engineering Department, The University of Jordan, Amman, 11942, Jordan; b School of Mechanical Aerospace and Automotive Engineering, Coventry University, United Kingdom

**Keywords:** Agent-based simulation, VRP-based GIS model, Word-of-mouth, Customer satisfaction, Parcel delivery logistics

## Abstract

The quality of Third-Party Logistics (3PL) services represented by delivery time decides the outcome of customer satisfaction. The result of this satisfaction judges the type of Word of Mouth (WoM) that, if positive, plays a vital role in attracting non-customers who are willing in 3PL services to join as customers.

In this paper, we investigate the effect of an essential factor represented by Word of Mouth on the number of customers in 3PL companies. Therefore, an agent-based model for parcel delivery is developed to investigate the impact of social factors such as WoM and other operational factors, including vehicle number and speed, on customer number and satisfaction, average service time, and vehicle utilization.

As a methodology, state charts of Vehicle, Customer, Hub agents are developed to mimic the messaging protocols between these agents under the WoM concept. A case study based in 3PL in Jordan is used as a test bench of the developed model. A sensitivity analysis study is conducted to test the developed model's performance, including different levels of influential model parameters such as targeting non-customers parameters by Loyal/Unhappy customers.

Key results reveal that the best scenario is achieved when the WoM value equals 10, the vehicle number equals 30, and the vehicle speed equals 60 km/h. These model parameters result in higher customer numbers of 873, vehicle utilization equals 63%, and customer satisfaction equals 99%. Video of our proposed model showing it in action can be found at: https://www.youtube.com/watch?v=3rR4l130-QU.

## 1 Introduction

The service industry has grown dramatically regarding service time, cost, and quality, to keep pace with technological advancement and customer shopping behaviors. As a result, companies in the service industry actively look for new opportunities to save time and money while delivering products to customers on time. Therefore, delivery service has become one of the service industry's pillars and has recently received a significant boost in the research literature.

Delivery service includes many vital parameters for the service industry, such as vehicles' availability, allocation of tasks, quality, and delivery time reliability. Moreover, a cheap yet reliable and fast delivery can be accomplished by taking advantage of autonomous vehicles interacting with Hub and Customers. Therefore, customer satisfaction and loyalty can be increased.

Autonomous vehicles are set to make a fundamental change to the transport business – and it will not be long before this happens. Autonomous vehicle technology will also enable entirely new delivery concepts, particularly for inner-city areas, as claimed and illustrated in research papers published by two leading logistics companies, DPD and DHL. Some applications of autonomous vehicles used in delivery service include, but are not limited to, support vehicles for letter and parcel deliveries, mobile repositories or pickup stations [1], and complete flexibility with autonomous courier vehicles [2].

Deploying autonomous vehicles in the delivery service industry may result in some problems due to the complexity of task-allocation algorithms, including unanticipated situations such as failure(s) of vehicles. Such issues can be prevented by using machine learning based on predictive and preventative maintenance. Therefore, the delivery service problem is challenging to model due to the complexity of the model's parameters.

Avoiding such problems would be of great concern for any leading-edge company. Attempting improvement scenarios on the existing system might be problematic regarding time and cost, including a certain amount of risk. Making a mistake during the experiment could be irreversible because building and destroying experiments might be unaffordable. Moreover, updating the investigation might be troublesome or even impossible once the experiment is done. However, understanding the system, predicting its challenges, and visualizing it can be done through simulation.

Simulation has lately come into the real world, enabling companies to model real-life problems without physically applying them in reality, with a specific level of abstraction. Important details can be added during the simulation process, whereas unimportant information can be left aside at any time. There are mainly three methods of simulation, namely: System Dynamics (SD), Discrete-Event Simulation (DES), and Agent-Based Modeling (ABM).

ABM is a relatively new method compared to SD and DES. It may provide insights into how the system's objects behave. It allows identifying the active system entity (agent), assigning its behavior logic in an environment (agent environment), and connecting to other agents (agent interaction). An entity may have some, but not necessarily all, properties to be an agent, such as proactive and reactive qualities, spatial awareness, learning, social interaction, etc. Agents can represent vehicles, people, products, orders, units of equipment, ideas, etc. The macrostructure of events in the system is determined by the agents' actions, as they decide their active state. Furthermore, they can decide on their current state depending upon their defined logic and characteristics [3].

One of the most critical points in the delivery service is customers' general satisfaction and perception. Since each customer has decision-making and behavior in the service industry, customers can be modeled as agents interacting, such as conducting the Word-of-Mouth (WoM) phenomenon. Another critical point is to reduce the time or cost of operations or both. The objective of minimizing the time and cost for the distance traveled can be accomplished with an effective interaction between autonomous vehicles. This interaction can be appointed by characterizing every vehicle in the model as an agent, communicating with the other vehicles and the retailer simultaneously to check their behavior logic.

Researchers have made many attempts to implement best practices in the service industry, such as the retail industry. Unlike earlier studies, this research focuses on delivery services in a local and short-haul domain. Notably, it is assumed that a Parcel Delivery Company will deliver parcels to its customers using autonomous automobiles (driverless vehicles). Each customer group is in a different region, and each customer has a different satisfaction level based on their feedback and the purchase repetition behavior. The company is assumed to centralize its central hub in an optimal location, depending upon how the city's population is distributed in different regions. The main interest of this research is the customer satisfaction effect on the delivery service.

Particularly as of the studied system, ABM framework is emphasized in modeling the behavior of individual customers/possible customers, parts of the service seller's resources, communication across or within different groups of agents and decision-makers in the system, as well as reactions of the system to the change of parameters and the exchange of the information. This study investigates the minimum required autonomous vehicles to achieve high customer satisfaction by considering the WoM effect on the population.

Given the various agent-based models being adopted to solve VRP problems, this paper aims to develop an agent-based GIS model to solve parcel delivery problems under the VRP category using autonomous vehicles. It investigates the effect of WoM on the number of current customers of the company. The main contributions are:

• To propose an agent-based GIS model for autonomous vehicle routing.
• To present new state charts related to agent-based GIS models.
• Introduce social parameters such as the WoM and other operational factors, including vehicle speed and the number of vehicles.

This paper's contribution is beneficial for logistics and transportation planners in managing delivery and collections services by understanding the benefits and issues related to the agent-based GIS models in optimizing their operations.

The rest of this paper is organized as follows: the first section will present related studies in which the gap in knowledge covered in this work will be discussed. The primary framework model with details about the agents' paradigms is described in the methodology section. Then, a case study is presented in the conceptual modeling section. After that, a discussion of the study results and computations are included in the results and discussion section. Finally, conclusions and future work will be given.

## 2 Literature review

This research focuses on the logistics service domain through studying the performance of parcel delivery company systems from the perspective of VRP. Several researchers used Agent-Based Simulation to solve VRP-GIS problems, including but not limited to [4], who worked on VRP with asymmetric costs to propose new realistic sets of model benchmark instances. They focused on the urban distribution system and used the T map API for Korea's real-time path analysis and distance measurement. Another work done by [5] aimed to develop an Agent-Based Simulation model to study the effects of urban logistics schemes on multiple actors. Vehicle Routing Problem of grain logistics based on GIS study was conducted to reduce logistic distribution cost [6, 7].

Authors [8] performed the VRP model into GIS for a logistics distribution. First, they applied spatial clustering to divide customer locations based on population density. Then, the optimum route for each customer location was achieved using the Ant Colony algorithm for a better solution. Our current work investigates delivery routing systems for a logistics company using the VRP-GIS model under the effect of complex factors like WoM, different vehicle speeds, and fleet size. Similar work was done by [9]. They presented a spatial parallel heuristic approach for solving large-scale VRPs. Authors [10] used the Agent-Based Modeling and Simulation (ABMS) approach to simulate Dynamic Vehicle Routing Problem (DVRP). They used GIS data and Particle Swarm Optimization to mimic retailers' location and road infrastructure in the existing system and evaluate optimal routes. Experiments were conducted to assess the effect of the degree of dynamism on logistics performances.

Authors [11] implemented a VRP-based GIS model using ArcMap's network analysis tool to minimize transportation costs and balance the workload. They considered dynamic traffic conditions, time windows, vehicle capacity, and driver working hours [12]. Used two spatial network tools, namely, "PhotoTracker" and "ArcGIS," to record and analyze vehicle routes in a day-care center.

Authors [13] developed an Agent-Based simulation model to solve the inventory routing problem considering the effect of WoM and shortages of distributions points demands on operational decisions. The influence of WoM communication on consumer behavior was studied by [14]. They found that there is a strong relationship between WoM and consumer attitude. We have considered WoM in our current research as a potential factor that affects the parcel delivery service company's number of customers.

Authors [15] provided an extensive literature review focusing on sustainability practices in urban vehicle routing that incorporate economic, environmental, and social concerns. The main finding from their study is that the prominent driver of sustainability among the three pillars is the economic dimension. Our current research focuses on the social dimension via the so-called "WoM" as the primary factor affecting the logistics company's market share.

Authors [16] presented a GIS-based optimization method utilizing a Tabu search algorithm for a Multi-Trip Heterogeneous Fixed Fleet VRP. The model stores, analyze, and visualizes all data and model solutions in geographic format. Various parameters, such as demand, number of vehicles, vehicle speed, and capacity, were considered. Our model has some of these parameters in addition to the WoM [17]. also integrated GIS and Tabu-based optimization for solving VRP with loading and distance requirements.

Another excellent example of applying ABMS in the transportation field is proposed by [18] by developing an Agent-Based Simulation (ABS) tool that allows vehicles' modeling movements from one depot to several customers based on two routing strategies. The shortest path routing algorithm was used to optimize the distance. In contrast, LANTIME optimized the CO_2_ emission by considering the expected vehicle's speeds in different road segments and different timeframes. The work done by [19] is another example of utilizing Agent-Based Simulation to develop a model of overnight parking choice for commercial vehicles.

The use of automatic guided vehicles to move goods in automated warehouses was studied [20]. They utilized Agent-Based simulation to analyze the behavior of automatic logistic warehouses considering specific factors.

From the previous literature, it has been noted that only a few researchers considered social factors such as the WoM factor and its impact on different operational decisions in other problems such as inventory management problems other than the VRP-GIS problem. Moreover, this work introduces an Agent-Based GIS model to solve VRP problems, mainly parcel delivery-based GIS environments, considering the effect of WoM and other factors such as the number of autonomous vehicles and their speed on the logistics operational decisions.

## 3 Development of the parcel delivery system

The world in which agents exist and interact and respond to static and dynamic variables and parameters in the system includes agents themselves, houses, routes, the flow of information, and the hub. Four of them represent the focus entities of the system: Vehicle, Customer (or non-customer), Hub, and the Main.

Usually, each of the company's delivery vehicles starts at the Hub. That is, as a matter of initial location. Since the vehicle's capacity is assumed to be four packages, it anticipates allocation with that number as a maximum for the orders it can be assigned in one task. The vehicle starts its delivery task as soon as four orders are allocated. If less than four orders are given for it within 1 h, it is triggered by a timeout to start its task to avoid long delays. As for the delivery task arrangement, the vehicle determines its first destination out of its assigned customers to be the nearest by the route. The remaining locations, if any, are compared at each customer location where the vehicle arrives. This approach is considered more efficient, especially in cost, than moving to the multiple customers' sites in an order that depends on the distance merely from the initial point, as two customers could be near. At the same time, a remaining third is nearer than one of them to the Hub. Finally, the vehicle returns to the Hub, which anticipates allocating another task, and the same logical course is repeated.

The beneficiary logistics companies of this model include Third-Party Logistics (3PL) on behalf of other companies such as retailers and manufacturers. These companies target customers, including households, small companies, businesses, medical centers, labs, etc. Parcels are the only product this 3PL company delivers.

The company divides people into customers and non-customers who could become potential customers as time advances. Initially, the company starts advertising as it has no customers initially; thus, some non-customers become customers and start ordering their parcels from the company, and so on. Advertising is not the only factor that affects non-customers to become potential customers. An indirect marketing method named WoM, an extra element describes how customers pass on verbal feedback to other people, who have a certain probability of changing their status towards the company depending on their perception, depending on customers' satisfaction/dissatisfaction. The customer sends an order to the company; the company allocates their order to any vehicle in the Hub; otherwise, if there were no available vehicles in the Hub, the customer waits until a vehicle returns to the Hub able to fulfill the order. However, the intermediacy of the company between the customer and the vehicles was skipped. This assumption simplifies the way of how tasks are allocated. The level of satisfaction determines whether this customer would like to order once again or become a non-customer, depending on the duration of parcel delivery fulfillment.

### 3.1 Conceptual modeling

**Figure 1:**
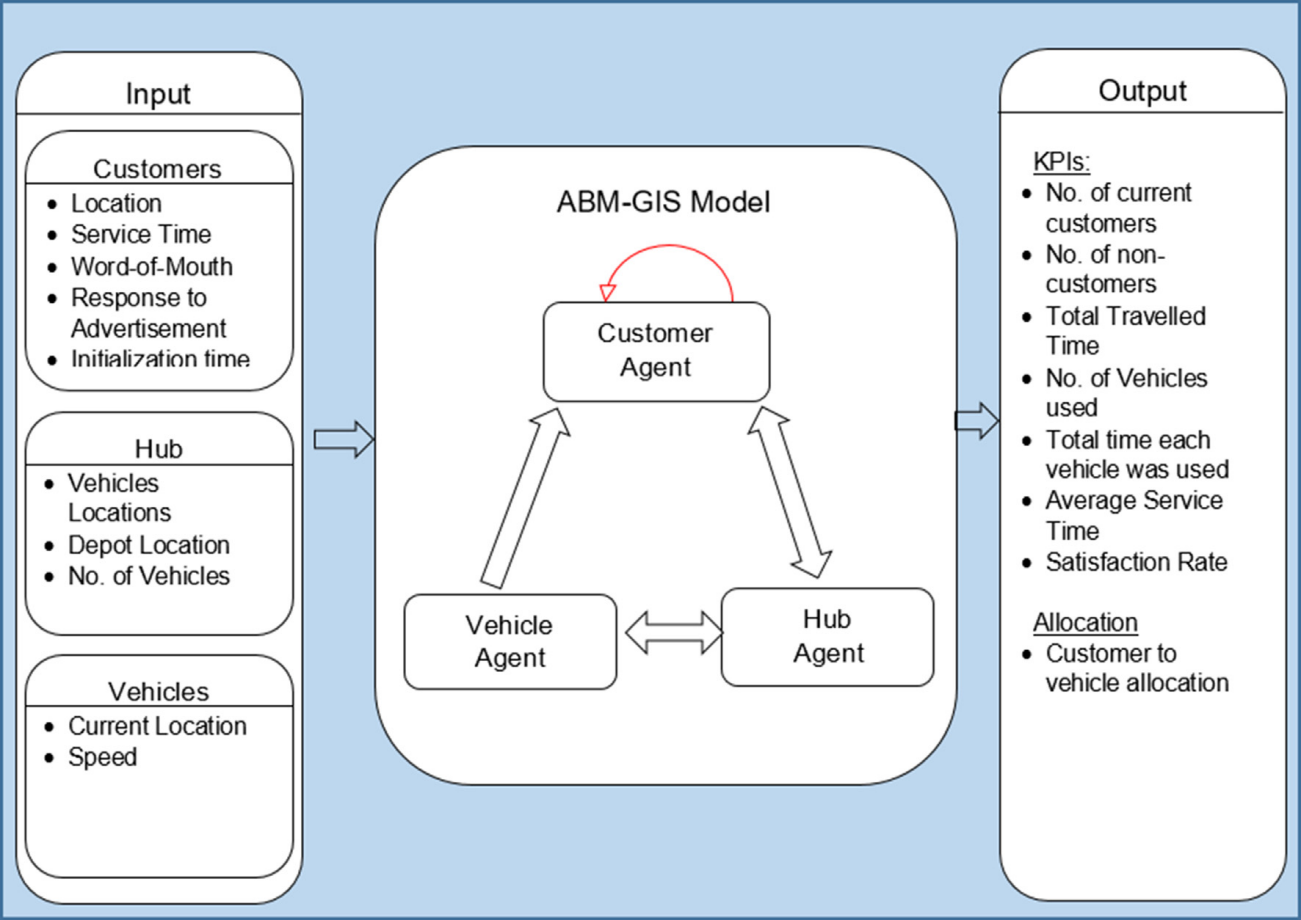
VRP GIS-based ABM architecture.

The purpose of developing the VRP model is to simulate then achieve the best parcel delivery process. Thus a model architecture is designed to demonstrate the parcel delivery model's inputs, processes, and outputs. See Figure 1 for the VRP model architecture.

In Figure 1, the input data is all the customers and vehicles where they can be initialized as agents. These data will then set the initial attributes for these agents. As a result, customer agents will have these attributes: location, service time, WoM, response to the advertisement, initialization time, and if current customer or not. In contrast, a vehicle agent has the current location, speed, capacity, current location, and total mileage. On the other hand, the desired model outputs should be vehicle routes measured against specific objectives: number of customers, total traveled time, number of vehicles used, total utilization time for each vehicle, average service time, and satisfaction rate.

Figure 1 illustrates how the core modules, including Customer, Vehicle, and Hub communicate. The Customer agent stores its attributes, for example, location and initialization time, and provides them when requested. The Vehicle agent has specific tasks, including communication with the Hub to receive orders sent to the Hub by customers. The Vehicle will let the Hub know its current attributes, specifically, location and current orders assigned. The Hub then decides if this Vehicle should be assigned to the current received order based on the Vehicle location and left available capacity. In addition, the Hub agent also controls the advertisement plans based on the percentages of current customers. It communicates with customers based on the advertisement plan to attract non-customers accordingly. Also, customers communicate with each other to represent the social effect of WoM.

Variables are generally used to store the results of model simulation or model some data units or object characteristics that change dynamically. The parameters used in the model are represented in Table 1.

**Table 1:** List of parameters.

Parameter	Description	Unit
*number_non*	A dynamic counter variable that counts the number of non-customers	Number
*numberCustServd*	Keeps track of the number of parcels that have been delivered	Number
*numberOfTrips*	Keeps track of the number of trips that the Vehicle has completed so far	Number
*IDaccumulator*	Is responsible for giving the customer order its ID number	Counter
*IDtoBeServed*	Identifies the customer to be served	ID
*totalTime*	Stores the cumulative value of trip durations	Hour
*maintenanceCost*	Stores the cumulative value of maintenance cost	$
*totalMileage*	Stores the cumulative value of the distance that has been traveled by all vehicles	Miles
*numberCar*	Determines the number of vehicles the company has	Number
*numberCar*AtHub	Keeps track of the number of vehicles that exist in the Hub at any time	Number
*numberOfTrip*	counts the number of times the vehicle has left the Hub	Number
*totalCumulativeUtilization*	stores the cumulative value of utilization for each trip that has been completed	Percentage
*timeOfStartingTheTrip*	this variable takes the value of the current simulation time upon exiting the State AtHub as it starts the task	Hour
*timeEndOfTrip*	this variable takes the value of the current simulation time upon arrival at the Hub at the exit of the State ToHub	Hour
*totalTripTime*	this variable stores the difference between values of the two variables mentioned above to record the total task time up until returning to the Hub	Hour
*cumulativeTripTime*	this variable stores the cumulative aggregation of all task times for a vehicle	Hour
*currentUtilization*	this variable is updated after completing each task, with the cumulative task time for the vehicle divided by the total simulation time; *currentUtilization* = *cumulativeTripTime/time()*.	Percentage
*avgServiceTime*	Average service time (all orders)	Hour
*numSatisfied*	Represents the total number of satisfied customers	Customer
*numNonSatisfied*	Represents the total number of unsatisfied customers	Customer
*main.houses.size()*	Represents the number of all customers	Customer
*order*	Represents the number of orders	Order

All the involved modules presented in Figure 1, including Vehicle, Customer, and Hub, will be explained in detail in the following sections.

### 3.2 The vehicle module

**Figure 2:**
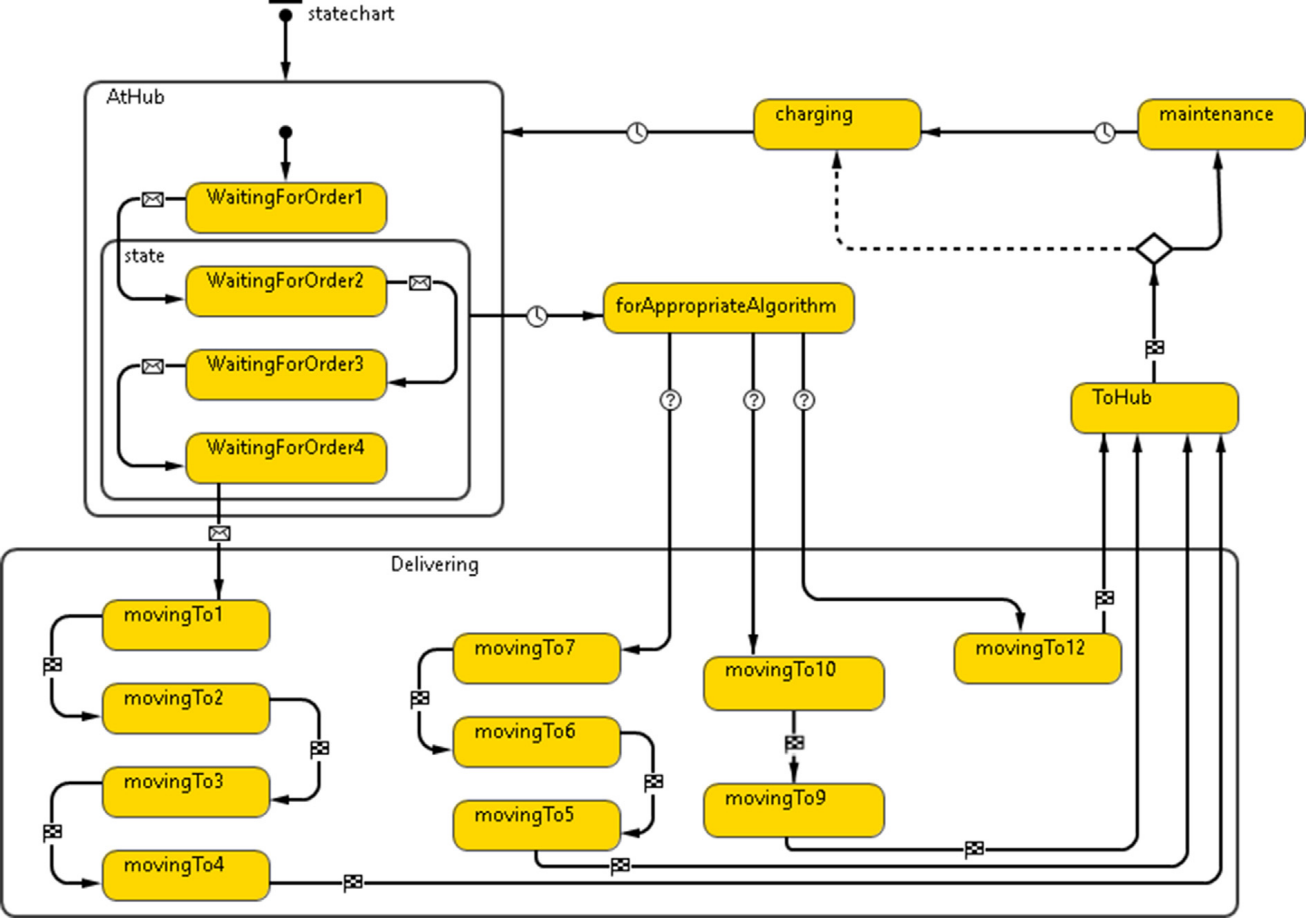
Vehicle statechart.

This module mimics vehicles as an agent used in the Parcel delivery process. It allocates vehicles to customers considering both location and vehicle capacity. A Statechart is developed to describe vehicles' behavior and communications with other agents. It also defines the established communications with other agents (Customer and Hub) in the VRP model introduced in the same environment. Figure 2 represents the Vehicle Statechart.

In Figure 2, the first State of the vehicle is defined to be *AtHub*. Multiple identical states are placed. The first is *WaitingForOrder1*, where the vehicle anticipates being assigned the first order of its complete upcoming tasks. When it is assigned its first order, it is triggered by receiving a message to activate the next State, *WaitingForOrder2*. The same logic is applied until the assigned vehicle reaches its maximum capacity. However, the number of actual orders is limited by the time passes after the first order is assigned to the vehicle. A Timeout trigger forces the vehicle to start the task if it is not filled to its maximum capacity within 15 min.

The vehicle then activates the Delivering State, which contains four options corresponding to the different possibilities as to how many orders it will deliver. It either has one, two, three, or four orders, depending upon the number assigned during the 15-minute Timeout window starting after the first assignment. Logically if less than the maximum capacity is assigned to the vehicle, the group of consecutive states to be activated is determined instantaneously before entering the complex State in a Branch module. Then, one of three mutually exclusive transitions is transitioned through depending upon a condition relating to the value of an internal variable named *numberOfOrders*. The value of this variable is initially 0 and is increased by one after each instance of the vehicle's receiving an assignment message.

Regarding customers' locations and referring to the vehicle Statechart, Delivering State, the distance to each assigned customer by GIS route is stored in a corresponding variable (distance1, distance2, distance3, or distance4), the minimum distance is stored in a variable named distanceR. An if-statement then determines an order to be the first to be delivered if the distance to its corresponding customer is equal to *distanceR*. The vehicle moves to the given customer's location by the function *moveTo()*. The below code, Logic Code 1, is an example of one of the moving orders logic in the Delivering State.

Logic Code 1 Vehicle "Moving To"

distance1 = distanceByRoute(order1.customer);
distance2 = distanceByRoute(order2.customer);
distance3 = distanceByRoute(order3.customer);
distance4 = distanceByRoute(order4.customer);
distanceR = min(min(distance1, distance2), min(distance3, distance4));
main.totalMileage = main.totalMileage + distanceR;
mileage = mileage + distanceR;
**if** (distanceR == distance1){
  moveTo(order1.customer);
  send("onway", order1.customer);
  distance1 = 100000000;}
**else if** (distanceR == distance2){
  moveTo(order2.customer);
  send("onway", order2.customer);
  distance2 = 100000000;}
**else if** (distanceR == distance3){
  moveTo(order3.customer);
  send("onway", order3.customer);
  distance3 = 100000000;}
**else if** (distanceR == distance4){
  moveTo(order4.customer);
  send("onway", order4.customer);
  distance4 = 100000000;};

In Logic Code 1 above, the task mileage and the total mileage for all vehicles are updated by arithmetically adding *distanceR* to the variables mileage and *main.totalMileage*, respectively. Upon arrival at the destination, the vehicle sends a message "arrived" to the customer to inform them. Then the next State is activated where distance comparison is carried out again, after determining the distances to the remaining customers' locations from the current point. The Vehicle calculates the distance from the Hub to each of their delivery points, selecting the shortest path and moving to the associated delivery. In equidistant deliveries, priority is given based on their order in the list.

The State *ToHub* is activated after the delivery task is completed, designated as the State. The vehicle moves back to the Hub by calling the function *moveTo(main.Hub)*. The variables mileage and *totalMileage* are updated again by the distance to the Hub. The variables designated for the distances and the task mileage are reset, as well as those designated for orders' information. A Branch module then determines whether the vehicle directly starts the charging process or goes through maintenance first, depending on the Boolean value of the condition *mMileage > 5000000*. This variable *mMailage* value represents the vehicle's mileage (used to check if maintenance is required), is updated upon arrival to the Hub, and is reset each time regular maintenance is applied. The Maintenance state represents a delay as an exit from it is triggered by a timeout given by two-parameter *exponential distribution (0.067, 5) minutes*. This distribution has been used extensively in reliability, maintenance, and survival analysis, and hence it was selected. The Charging timeout after each completed delivery task depends on the task's mileage. The Vehicle has a 24 kWh battery, consuming 0.0002 kWh per meter, and charging the battery 1 kWh takes 1.25 min; thus, the timeout is given by Eq. (1).

(1) Timeout=0.0002×Mileage×1.25+2

After charging, the vehicle finally returns to the complex State *AtHub*, indicating its availability to be assigned new orders.

### 3.3 The customer module

**Figure 3:**
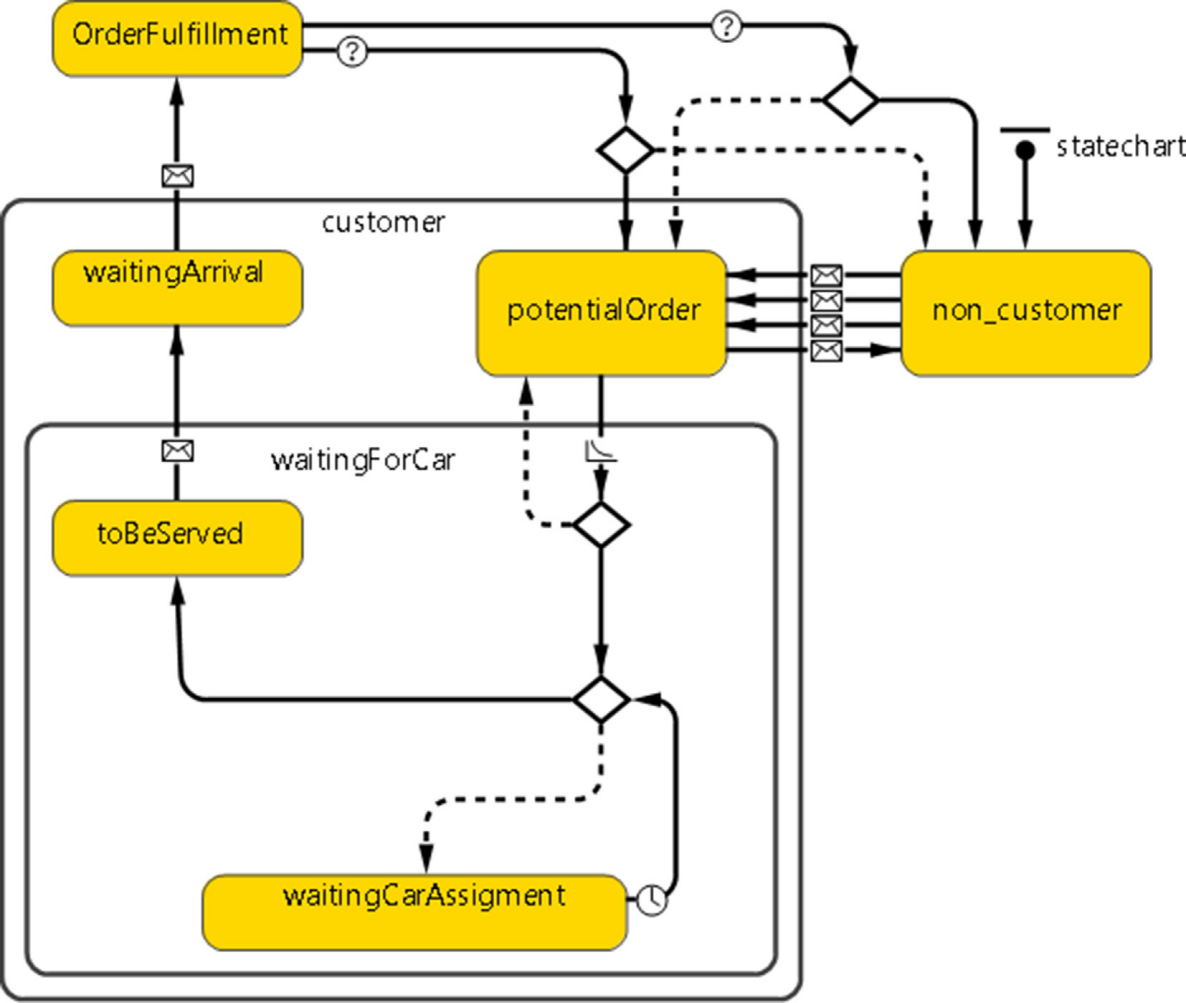
The customer statechart.

This module mimics the Customer as an agent used in the Parcel delivery process. It defines all states and communications with other agents (Hub and Vehicle) and the customers themselves. Based on customers' satisfaction driven by the time waited, customers will spread the word about the transportation company positively or negatively. Figure 3 represents the Customer Statechart.

In Figure 3, the Statechart starts with a non-customer state. This state represents the initial place that the customer lives in, and they are all initially not defined as customers to the delivery company. This state has four ways:

● A strong marketing campaign from the company can result in a decent advertisement that makes the non-customer order from the company. The transition is done by sending a message to the customer that says, "*come.*" The sent message has a probability *P(Attracting a customer given Strong Advertisement)* to target any random non-customer from a whole population.
● A normal marketing campaign from the company can result in an intermediate advertisement that makes the non-customer order from the company. Here, the transition is also done by sending a message to the customer that says *"comeifyouwant."* The sent message has a probability *P(Attracting a customer given a normal advertisement)* to target any random non-customer.
● Loyal customers to the company that gives a good review for the service, Namely, the WoM effect, might make non-customers order from the company. The transition is done by sending a message to the customer that says "*cool.*" In contrast, the message sent has a probability of *P(targeting random people given happy customers)* to target random people from a whole population.
● Unhappy customers about the company service give a negative review for the service, Namely, WoM effect, that might make customers not order from the company. The transition is done by sending a message to the customer that says "*not-cool.*" In contrast, the message sent has a probability of *P(targeting random people given unhappy customers)* to target random people from a whole population.

Each order has an ID and time of initiation that is concerned with a variable to account into display statistics. The order then goes into a decision-making Logic Code 2, either allocating the order to the Vehicle if at least one available Vehicle at the Hub or waiting until a Vehicle becomes available.

Logic Code 2 Customer Order Allocation

*main.IDtoBeServed+ = 1;*
*if (truck! = null)*
 *send(order, truck);*
*shapeBody.setFillColor(green);*

The variable *toBeServed* is increased by one if the order has been initiated. A check on the Vehicle's availability is done to assign the vehicle or wait for one to be available. The customer's color is changed to green showing that he is waiting to be served. Then the Vehicle sends an "*onway*" message to the customer who ordered. Thus, the customer is transitioned to "*WaitingArrival*," considering that every transition has its visual color to monitor changes easily. Upon the order's arrival to the customer and order fulfillment, the transition that leads to this State is done by a customer message saying "*arrived*."

The customer's perception is determined by the differentiation between the total time and the time tolerance that the customer can wait within, if it is within tolerance, so the customer is guaranteed a second time order. If not, the customer becomes a non-customer again, both done by a condition transition as shown in Logic Code 3. The variable *totalTime* tallies the total time of the service (time between order initiation and arrival to the customer).

Logic Code 3 Customer Perception

*If totalTime > normal((1.5/60), 1)*
  **for**(**int** i = 0; i < main.wordOfMouth; i++)
  sendToRandom("not_cool");
*If totalTime < normal((1.5/60), 1)*
  **for**(**int** i = 0; i < main.wordOfMouth; i++)
  sendToRandom("cool");

Firstly, suppose *totalTime* for service is larger than the value specified by the normal distribution with a mean of 1 h and standard deviation of (1.5/60) hours. In that case, the customer becomes non-customer back again. This condition results in sending bad feedback to other people, sending a "*not_cool*" message to a customer who was willing to commit an order, leading them to change their mind and become originally a non-customer. The second if statement does the opposite when the *totalTime* for service is less than the value specified by the same normal distribution, resulting in second time order. As mentioned earlier, the customer who prefers the company's service will send a message "*cool*" to several random persons, denoted as the WoM effect.

### 3.4 The hub module

**Figure 4:**
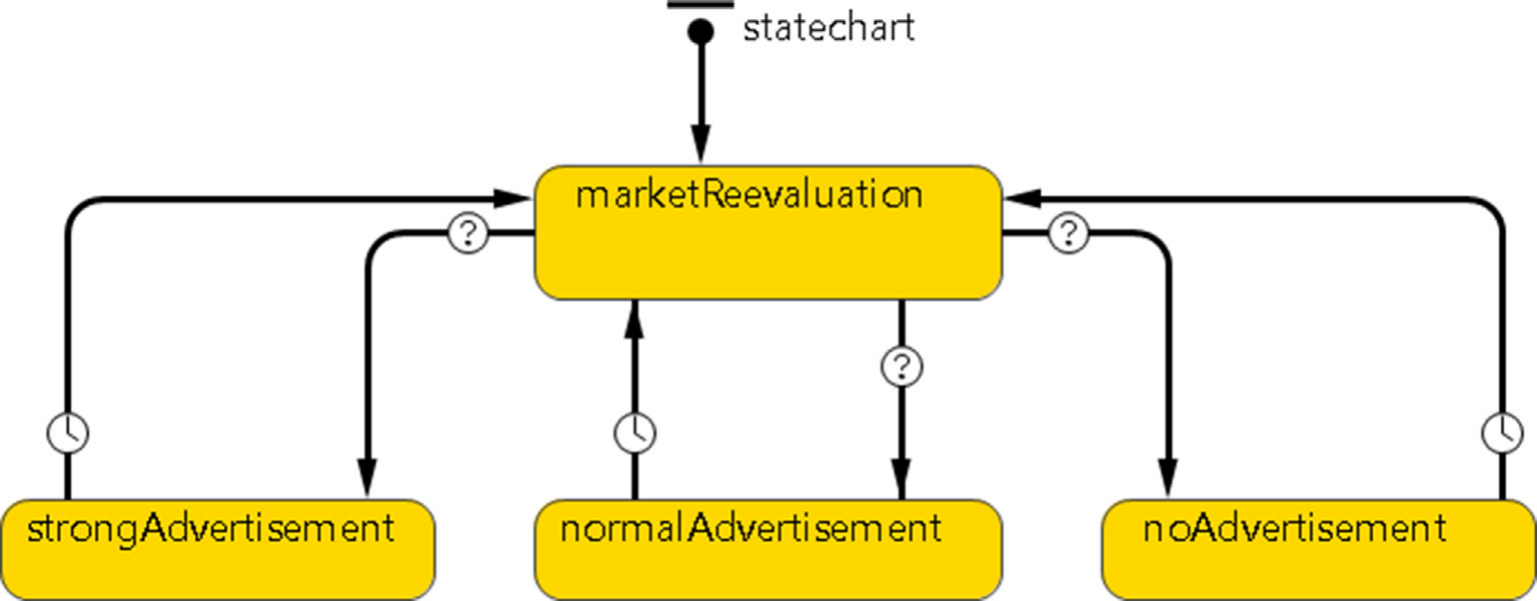
The hub StateChart

The agent Hub represents the company and its location. The Hub is a system of workers responsible for communicating with vehicles and customers. However, the main role of the agent Hub remains one representing the company's marketing-related decision-making, particularly in terms of advertisement campaigns and how they are managed. It manages the marketing campaign, which depends on the percentage of current customers. See Figure 4 for the Hub StateChart.

In Figure 4, the first Hub state is defined as "*marketReevaluation,"* through which the percentage of current customers is updated each time a change happens on the "*non_customers"* variable. The second state, "*strongAdvertisement*" defines the strong advertising plan that the company will conduct to attract more customers if the percentage of current customers, captured by the "*percentCustomer"* variable, has dropped below a predefined threshold value. The "*strongAdvertisement"* state does this by sending a "*cool*" message to all customers through the code *sendToAll("come");*.

A similar logic is applied for the "*normalAdvertisement"* state if the percentage of current customers is greater than a predefined lower level and less than a higher level. The "*normalAdvertisement*" state does this by sending a "*comeifyouwant*" message to all customers through the code *sendToAll("comeifyouwant");*.

## 4 Case study

### 4.1 Company brief

The company selected for this study is Jordan's well-known logistics services provider. They are classified as a 3PL service provider. It has many hubs that cover most of the cities in Jordan. The company size in terms of fleet size is considered large. Parcel delivery and pickup are one of the main activities of this company. They have concerns about WoM and advertisement plans' effect on their business and performance, located in Amman, Jordan. The company approached the researchers working at The University of Jordan to evaluate the current business workflow and suggest other viable improvement scenarios to enhance their market share and customer satisfaction. The company provided all data, including actual locations of customers and the amount of demand.

### 4.2 Data collection

The company owns 45 vehicles; 41 of them are being used. As an average, the vehicle speed is 35 km/h while the maximum allowed speed is 60 km/h within cities. In addition, this company experiences a WoM rate equal to 2.

**Figure 5:**
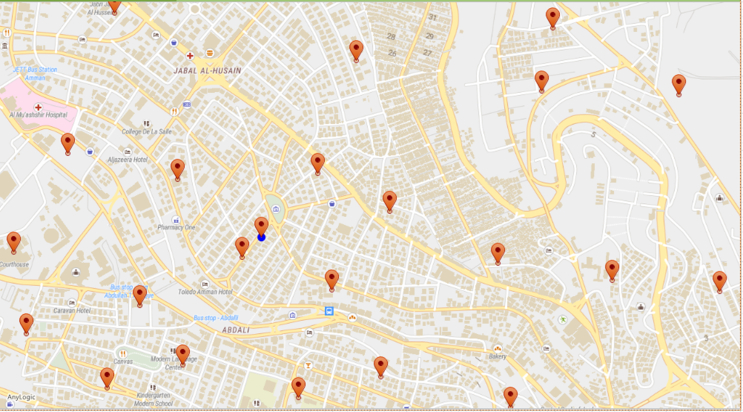
Sample GIS Map of the covered Regions in Amman, Jordan.

As mentioned earlier in section 4.1, the logistics company covers the cities include the following regions (Abdali, Ras ElEin, Madinah, Zahran, Yarmouk, Badr, Tariq, Marka, Naser, Basman, Qweismeh, Khreibeh, Yadoudeh, Um Qseir, Jubeiha, Sweileh, Tila, Khalda, Summaq, Abu Nuseir, Shafa Badran, Wadi Sir). See Figure 5 for the GIS sample map of the covered region by the Logistics company based in Amman, Jordan.

In total, 896 GIS points represent the total number of the selected customer in this case study. These customers all need the same service, either parcel delivery or pickup, and they can be any of the following types: Households, business companies, medical labs, and manufacturers.

The number of customers attracted in response to the advertising campaigns that the company has conducted was collected from their historical records. The company provided these numbers, which frequently appear for their annual forecasting and planning. The accuracy of these numbers is attributed to the company, and the authors have no control over the collection of data methods. Table 2 represents the advertisements response probabilities for customers.

**Table 2:** Advertisement response and customer states probabilities (As-Is).

Advertisement and Customer State	Customer Percentage Level	Message	Probability of Targeting Non-customer
Strong promotion Campaign	<30%	come	10%
Medium Promotion Campaign	30%–80%	comeifyouwant	5%
No Promotion Campaign	>80%	noadvertisement	0%
Loyal Customers	-	cool	50%
Unhappy Customers	-	not_cool	5%

In Table 2, two promotion campaigns types are run based on the customer percentage level. This level represents the current number of the company's customers out of the total potential customers in the market. For example, if the current number of the company's customers is less than 30% of the total number of potential customers in the market, the strong promotion, which includes Facebook, TV, Newspaper …etc., will be selected and run. If the current number of the company's customers is greater than 80% of the total potential customers in the market, there is no need to run a promotion campaign. Ultimately, the customer percentage level decides the campaign type.

The Strong promotion campaign will result in an attraction of 10% of the non-customers population. This ratio is good as these customers are new customers to the company, increasing the company's market share. The Medium promotion Campaign also attracts 5% new customers, and this compared to the budget allocated to such campaign is good.

WoM represents the number of non-customers that a customer (Loyal/Unhappy) is willing to share their experience with them. This factor is considered as an input and independent factor from any other parameters in the model, including Table 2.

For example, if WoM is 10, a Loyal Customer will spread the word and attract 50% new customers to the company, i.e., five new customers will join the company. In contractor, if the WoM is 10, an Unhappy customer might distract 5% of the current customers' interest in the company services and covert them to non-customers for this company.

**Figure 6:**
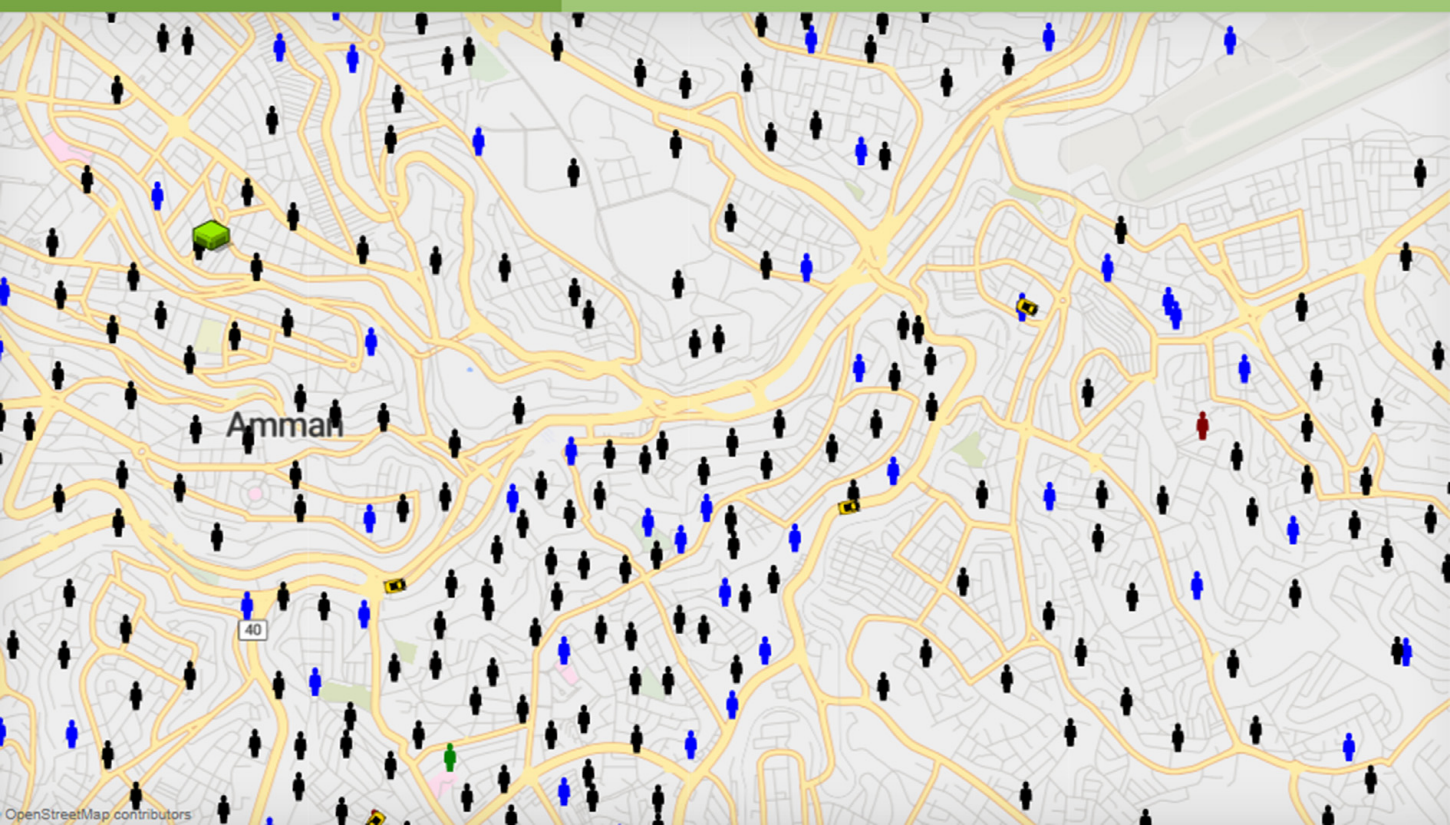
The Customer and non-Customer Representation in the GIS-Based Model.

Figure 6 represents customers' and non-customers representation in the ABM-GIS model.

### 4.3 Model verification and validation

The authors presented the model to the company to verify it. They walked through it together to discuss its functionality. The company's logistics team also reviewed all Statecharts, and they confirmed their applicability to the company workflow. In addition, the authors ran through the model and found and fixed a bug in the messaging system for the advertisements campaigns. An example of such a bug is that the message "*cool*" was coded instead of the "*not_cool*," which was fixed.

The validity of the range of the model parameters (Independent variables) used was considered based on the information provided by the company. These parameters are the Advertisement level, customer percentage level that triggers the promotion level, and WoM. The company has frequently studied their customers and successfully obtained initial values that we fixed and used in this model. The model output represented by the current number of customers and the percentage satisfied were relatively close to the company outputs. However, a sensitivity analysis study of the range of parameters is conducted in section 5 for further model validation.

### 4.4 Experimentations

The three factors (independent variables) are WoM, number of Vehicles, and Vehicle speed selected in three different levels for each. The minimum WoM selected was two because of the feedback from the company professionals. They also determined ten as the maximum that could happen based on their knowledge of the population. The number of vehicles actively used is 41, so we selected 30 as the minimum as a potential breakdown scenario and other scheduled maintenance services. The maximum number of vehicles is also tested, and this number was selected to be 90 as a possible future investment by the company to increase its fleet size. The Dependent variables (Responses) selected for this analysis are the Number of Customers, average Service Time, Number of Vehicles utilization, and Customers Satisfaction Rate. These were selected because they represent the system performance and their direct impact on the analysis and understanding the necessary improvement actions.

#### 4.4.1 Scenario analysis and discussion

This section aims to present the scenario results from the simulation model for the system KPIs in terms of the three factors mentioned earlier. The simulation model was run 50 times (number of replications), 9 h as the replication length (working day), and the average outputs for all scenarios are shown in Table 3.

**Table 3:** Experimental results (Number of replications = 50, Replication length = 9 h).

Scenario Number	Word of Mouth	Number of Vehicles	Vehicle Speed	Number of Customers	Average Service Time	Total Vehicles Utilization (by Number)	Percentage Satisfied
1	2	30	20	107	0.91	0.19	44.0%
2	5	30	20	464	0.83	0.80	57.0%
3	10	30	20	642	0.98	1.00	45.8%
4	2	60	20	119	0.88	0.05	46.4%
5	5	60	20	373	0.88	0.33	53.2%
6	10	60	20	780	0.80	0.77	61.8%
**7**	**2**	**90**	**20**	**87**	**0.94**	**0.03**	**37.0%**
8	5	90	20	419	0.86	0.24	52.3%
9	10	90	20	755	0.83	0.48	60.1%
10	2	30	40	354	0.50	0.40	85.8%
11	5	30	40	805	0.45	1.00	94.1%
12	10	30	40	865	0.42	0.87	96.5%
13	2	60	40	319	0.50	0.13	89.6%
14	5	60	40	791	0.47	0.42	91.4%
15	10	60	40	864	0.43	0.27	95.7%
16	2	90	40	277	0.49	0.06	87.8%
17	5	90	40	757	0.45	0.22	94.2%
18	10	90	40	866	0.43	0.28	93.4%
19	2	30	60	468	0.34	0.27	96.1%
20	5	30	60	843	0.32	0.50	99.1%
*21*	*10*	*30*	*60*	*873*	*0.31*	*0.63*	*99.2%*
22	2	60	60	406	0.36	0.17	96.8%
23	5	60	60	829	0.33	0.33	99.1%
24	10	60	60	877	0.31	0.32	99.1%
25	2	90	60	394	0.38	0.08	98.0%
26	5	90	60	835	0.32	0.17	99.0%
27	10	90	60	877	0.31	0.20	99.5%

It is evident from the results above that the best scenario is scenario 21, while the worst was scenario 7. Scenario 21 includes parameters WoM = 10, number of vehicles = 30, and vehicle speed of 60 km/h. It has a fairly high number of customers and high utilization relative to close scenarios. In addition, customer satisfaction is high. Other scenarios could have higher utilization; however, their number of customers is less, or the required number of vehicles is more. Thus, no dominating scenario can be found, but 21 seems to outperform others. This satisfaction is attributed to the combination of vehicle speed and WoM. An increase of both will increase the satisfaction rate to the maximum possible. On the other hand, increasing the number of vehicles will not affect the system performance, and eventually, sometimes, it just makes things worse. While the worst scenario is 7, which is mainly due to the low customer satisfaction achieved (37%), and very low utilization of vehicles (3%), and the lowest number of customers (87).

In general, increasing the number of vehicles will not have much effect since WoM is low, which results in not spreading the good word. On the other hand, increasing the WoM will help just if the number of vehicles is high, but for 30, it will help spread the negative experience of customers. It can also be concluded that the vehicle's speed is the main factor here, meaning that a low vehicle's speed will always result in higher service time and below acceptable customers' satisfaction rate.

It is worth mentioning that the loyal customers’ population is not fixed, as it depends on the level of satisfaction, i.e., higher satisfaction levels will result in more loyal customers (number of customers) and vice versa. This is included in the model as a relationship which affects the loyal customer population when more orders are been delivered on time, leading to higher satisfaction level. For example, for the WoM value of 5, the satisfaction level drops from 57% (Scenario 2) to 53.2% (Scenario 5), resulting in reducing the number of customers from 464 to 373, see Table 2.

#### 4.4.2 Impact analysis and discussion

**Figure 7:**
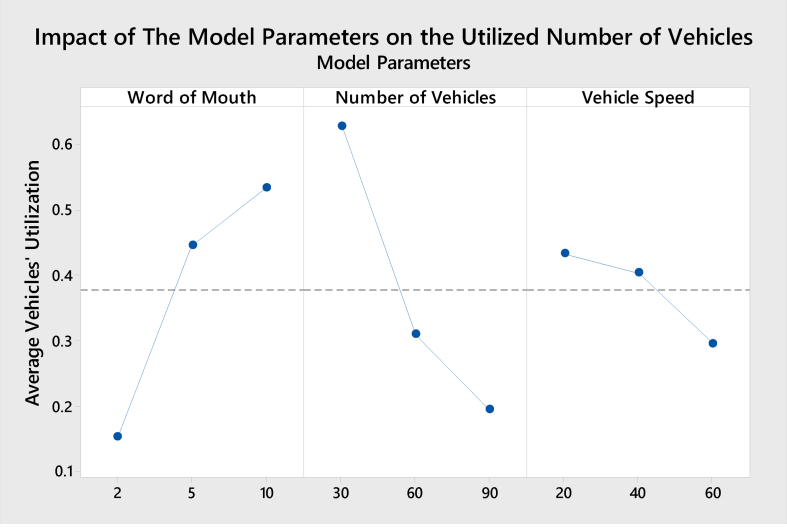
Main effects of model parameters on utilized No. of vehicles.

**Figure 8:**
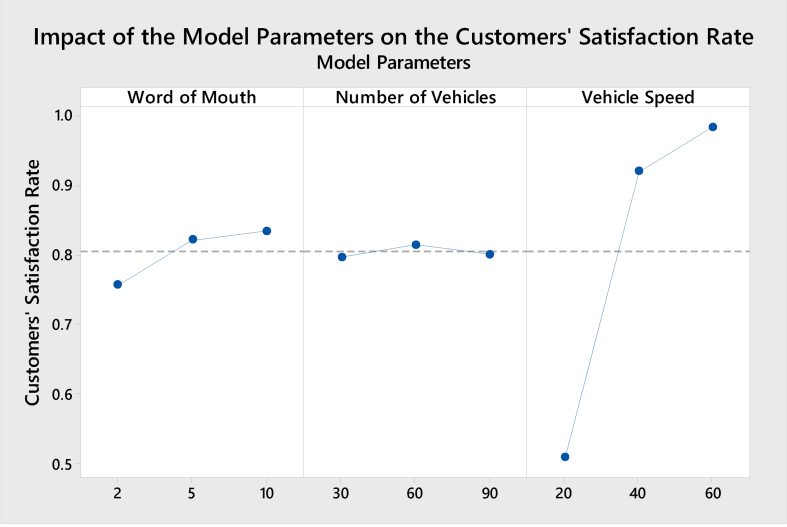
Main effects of model parameters on the customers' satisfaction rate.

**Figure 9:**
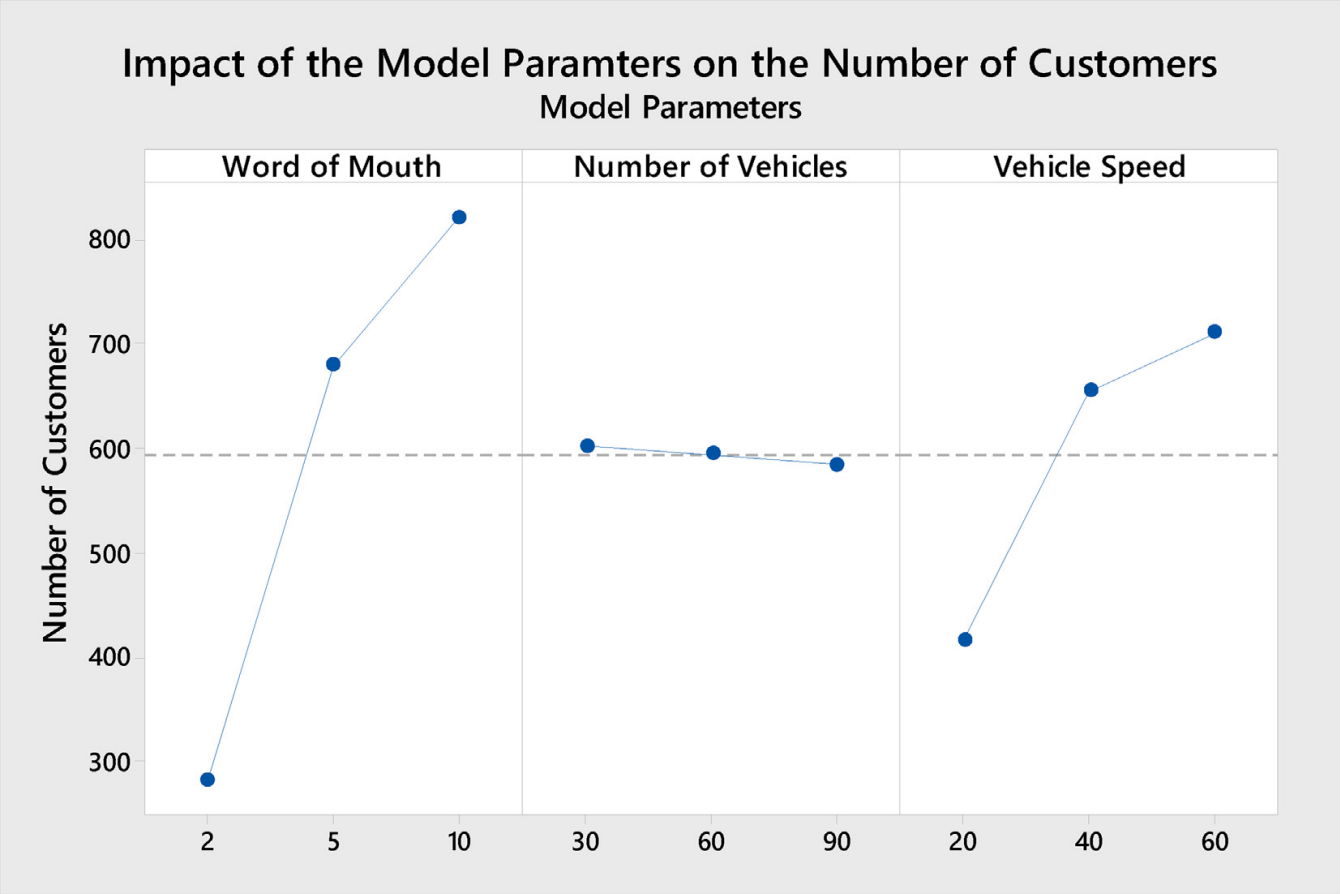
Main effects of model parameters on the No. of customers.

**Figure 10:**
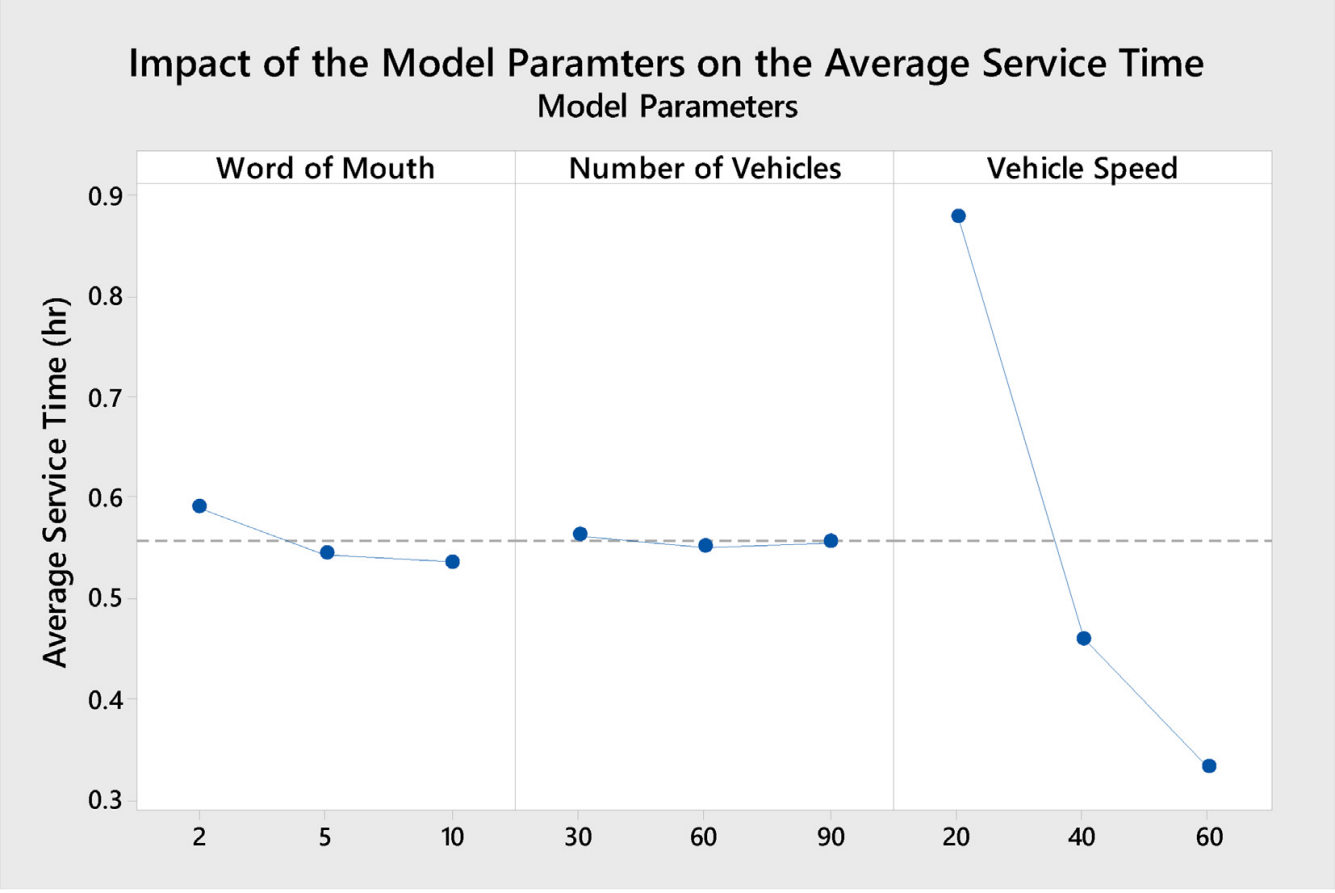
Main effects of model parameters on the average service time.

The purpose of this section is to present the impact of the model parameters on each of the model KPIs in terms of the number of customers, average service time, utilized number of vehicles, and average customers' satisfaction rate. The following Figures 7, 8, 9, and 10 show the results obtained from the Minitab software of the Impact for the model parameters on the four responses/KPIs. Figure 7 presents the impact of the model parameters on the average utilized number of vehicles.

All the parameters affect the utilization; as the WoM increases, the average utilized number of vehicles sharply increases accordingly. The reason is that when the WoM increases, the utilized number of vehicles increases, and that is because of the good reputation spread by the customers, which led to attracting more customers to the company. The utilization of vehicles decreases as the number of vehicles increase. This increase pushes the company to divide the parcel workload between more than one vehicle to comply with the code of practice - the safety of loads on vehicles [21]. Also, increasing the vehicle's speed will slowly decrease the number of vehicles utilized. This is to comply with the health and safety regulations of vehicles used in last-mile delivery [21].

When vehicle speed increases, fewer vehicles will be utilized as this number could serve the customer demand at the requested time. Figure 8 presents the impact of the model parameters on the average customers' satisfaction rate.

Increasing the WoM leads to an increase in customer satisfaction slowly. This is attributed to the increment in the number of happy customers who give a good feedback about the company's reputation. As discussed in the previous section, the number of vehicles has the minimum effect, evident from the stable relationship shown in Figure 8. The vehicle's speed has a significant relationship with customers' satisfaction, such that increasing the vehicle's speed will strongly increase satisfaction. Therefore, a good delivery experience influences repeat purchases, increasing customer satisfaction [22].

In general, increasing the WoM and vehicle speed will increase customer satisfaction. Figure 9 presents the impact of the model parameters on the average number of customers.

Increasing the WoM will significantly increase the number of attracted customers. This outcome is mainly due to the increase in the number of happy customers who give good feedback about its reputation and share their feedback with other non-customers. As discussed in the previous section, the number of vehicles has the minimum effect, evident from the stable relationship shown in Figure 9. The number of vehicles is independent, and hence different levels will not significantly impact the number of customers. Statistical analysis was made showing a P-Value equal to 0.992, indicating that the mean values of the total number of customers under the number of cars equal to 30, 60, and 90 are statistically similar. Even though it makes sense at the first glance that increasing the number of vehicles will result in increasing the number of customers; however, increasing number of vehicles does not affect the number of customers. The only influential factor on the customers’ satisfaction is the delivery time, which means that the shorter customer service time, the higher customer satisfaction. This shorter service time is achieved by current ongoing vehicles rather than additional vehicles released from their hub, which results in higher transportation time.

The vehicle's speed has a strong relationship with the number of customers. Increasing the vehicle's speed will significantly reduce the average waiting time of customers and subsequently attract more customers [22]. It is worth mentioning that the maximum speed allowed inside cities is 40 km/h. In general, increasing the WoM and vehicles speed will increase the number of customers. Figure 10 presents the impact of the model parameters on the average service time.

The most significant parameter that affects the average service time is the vehicle speed. As expected, increasing the vehicle speed will decrease the average service time. This is equivalent in practice to the fact that increasing vehicle speed will reduce average service time [23, 24].

On the other hand, the number of vehicles will not affect the average service time. This is because the number of customers is mainly affected by the other two factors, as discussed earlier. In addition, increasing the number of vehicles may help serve more customers, but vehicles still need to start the trip from the hub, which most of the time does not help in terms of the service time. Finally, the WoM slightly affects the average service time based on the results.

Happy customers will spread the positive feedback to a number of non-customers equal WoM. On the other side, unhappy customers will spread their bad experiences with the company through negative feedback. The aim was for fixed triggering levels on campaign levels, what is the effect of WoM on the company's share from total potential customers. One practical implication of the work is that the company can survey the market to observe the actual WoM. Based on that, it will decide on the number of vehicles and other parameters to maximize.

## 5 Sensitivity analysis

A sensitivity analysis study of changing model parameters is carried out to measure the changes of parameters' impacts on the outputs and verify the developed model's performance. It also monitors the relationship among these parameters. The impact of their variations in the model parameter settings is useful to identify the inputs that cause different KPI values for practical model analysis.

In this study, the behavior of the developed agent-based GIS model in response to different levels of Loyal and Unhappy customers to targeting non-customers for both the best and worst scenarios is investigated. The probabilities of targeting non-customers are presented in Table 2 and analyzed at different levels as it directly affects the company's total number of current customers. Table 4 presents the sensitivity study conducted to show the impact of different levels of targeting non-customer probability parameters on the total company customers for both the Best and Worst scenarios.

**Table 4:** Sensitivity of the simulation results to the probability of targeting non-customer parameters changes.

	Probability of targeting a non-customer by Loyal or Happy Customers	Best Scenario WoM (10), Number of Vehicles (30), Vehicle Speed (60) km/hr	Worst Scenario WoM (2), Number of Vehicles (90), Vehicle Speed (20) km/hr
		Total number of Customers	
Probability of targeting a non-customer by Unhappy Customer is 2%	25%	829	68
	50%	878	147
	75%	889	245
Probability of targeting a non-customer by Unhappy Customer is 5%	25%	820	59
	50%	877	87
	75%	885	199
Probability of targeting a non-customer by Unhappy Customer is 10%	25%	817	52
	50%	874	70
	75%	881	177

As shown in Table 4, increasing the Loyal customer's rate results in an increase in the total number of customers, while increasing the Unhappy customer's rate will decrease the total number of customers. For the Unhappy Customers, when the Loyal Customers is 50%, for example, it can be noticed that the total number of customers is increasing for both the best and worst scenario. This is attributed to the fact that increasing loyal customers from 25% to 75% will increase the total number of current customers in the company.

For example, in the best scenario, for the Loyal customer rate is 25%, the total number of customers will decrease from 829 when the Unhappy customer rate is 2%–817 when the Unhappy customer rate becomes 10%. In the worst scenario, it can be noted that the total number of customers increases from 52 to 177 when the Loyal customer rate increases from 25% to 75%, given that the Unhappy customer rate is fixed at 10%.

## 6 Practical implications

This study's results provide insight for logistics and marketing managers who must balance customer desires for convenience to increase their satisfaction with business desires, including resource allocation and investment for the best service efficiency at their 3PL companies. This subsequently will increase the total number of customers by attracting new ones through spreading word-of-mouth that has an influential impact on customer behavior [25] among other people who are willing in such services. It provides a tool for managers to address the trade-offs between various settings of the independent factors. This includes Loyal and Unhappy customers and their different rates that have been achieved at different satisfaction levels obtained by allocating other numbers and speeds of vehicles and the total number of customers to be served by 3PLs.

It also enables logistics planners to examine the effect of two factors of Loyal and Unhappy customers on the total number of customers to be served by 3PL companies. It also assists 3PL companies in generating the best plans for customer services. It identifies the optimal resource allocation plans needed to maximize customer satisfaction that will, in turn, increase the total number of customers as a consequence of increasing the rate of Loyal customers and decreasing the rates of Unhappy customers.

Ultimately, this study provides valuable guidance and insights for 3PL managers and marketing officers to understand how to guarantee the best customer convenience leads to increase their satisfaction to become Loyal customers. In addition, they will understand the effect of word-of-mouth exchanged between such Loyal customers and other people who are willing in 3PL services to increase the total number of customers. The same is applied and will be avoided when weak satisfaction leads to Unhappy customers who might exchange their practice through word-of-mouth with other people, which will reduce the total number of customers to be served by 3PL companies.

## 7 Conclusion and future work

This study aimed to develop an ABM-GIS model for the best parcel delivery practice. Statecharts of three agents, Customer, Vehicle, and Hub, were successfully delivered and modeled using the AnyLogic software.

The impact of three factors, including WoM (social), Number of Vehicles (operational), and Vehicle's Speed (operational), on the four performance measures/KPIs was investigated. These responses include Average Service Time, Customer Satisfaction, Number of Customers, and Vehicles' Utilization. A total of 27 scenarios were conducted to test the impact of the factors mentioned above on the model KPIs. The simulation was run for 50 replications with 9 h per replication to reduce error in results and achieve more accurate measures.

The results show that the WoM and Vehicle Speed were the most significant factors affecting the four KPIs. At the same time, the number of vehicles was substantial in the case of the utilization of the vehicle. Increasing the WoM will increase utilized vehicles, customer satisfaction, and Number of customers, but on the other hand, decrease the average service time. Increasing the WoM will bring happier customers who need more Vehicles from the number available ones at Hub, which justifies the increase in customers' satisfaction rate, the number of customers, and utilized vehicles. Also, more customers mean that vehicles will serve more than one customer on the way, resulting in less average service time.

Increasing the vehicle speed will decrease the utilization and average service time while increasing customer satisfaction and the number of customers. This outcome is straightforward to justify: the increase in vehicle speed will result in less total service time and less need for vehicles since, consequently, lower average service time and lower utilization. On the other hand, less average service time will increase the customers' satisfaction, encouraging them to spread the WoM and bring more customers, consequently increasing customers' satisfaction and number.

It has been concluded that the most economical and reasonable scenario represented by the best one was 21 (WoM = 10, number of vehicles = 30, vehicle speed = 60 km/h). It resulted in 99.2% customers' satisfaction, which is reasonably high, and a total number of customers of 873, which is also reasonably high, the average service time of 0.31 h, which is below the average value of the accepted time by customers provided by the normal distribution. Also, the utilization of vehicles is 63% which is relatively high utilization. The utilization is considered the distinguishing KPI here, making this scenario the most applicable one. The results of this paper were juxtaposed with results from previous literature. These results are related to the behavior of the obtained ones from the model of this study. In addition, these results were discussed in detail and linked with the related literature. Practical implications were discussed in detail to show how 3PLs could benefit from the developed model besides real-life applications. The limitation of this model is that it did not consider the competency and market share element that happens in reality.

As future work, the dependent variables (responses/KPIs) might be modeled using decision-making techniques such as multi-objective mathematical programming. This provides the company with a tool considering different KPIs to plan and make decisions on the optimal fleet sizing based on targeted customers' satisfaction, available investment budget, surveyed social WoM, and vehicles speed restrictions. In addition, the effect of different levels of advertisements and customer states could be investigated further. Therefore, the impact of campaign type (via Facebook, TV, Newspaper, etc.) on the current number of customers could be considered. Another research direction represented by identifying how many vehicles are needed for a given number of orders per hour if the hub is at different locations could be investigated.

## Declarations

### Author contribution statement

Mohammad Shbool and Ammar Al-Bazi: Conceived and designed the experiments; Performed the experiments; Analyzed and interpreted the data; Contributed reagents, materials, analysis tools or data; Wrote the paper.

Rami Al-Hadeethi: Contributed reagents, materials, analysis tools or data; Wrote the paper.

### Funding statement

This research did not receive any specific grant from funding agencies in the public, commercial, or not-for-profit sectors.

### Data availability statement

No data was used for the research described in the article.

### Declaration of interests statement

The authors declare no conflict of interest.

### Additional information

No additional information is available for this paper.
